# Bacteriological profile and drug susceptibility patterns in dacryocystitis patients attending Gondar University Teaching Hospital, Northwest Ethiopia

**DOI:** 10.1186/s12886-015-0016-0

**Published:** 2015-04-02

**Authors:** Yared Assefa, Feleke Moges, Mengistu Endris, Banchamlak Zereay, Bemnet Amare, Damtew Bekele, Solomon Tesfaye, Andargachew Mulu, Yeshambel Belyhun

**Affiliations:** Department of Ophthalmology, University of Gondar, Gondar, Ethiopia; School of Biomedical and Laboratory Sciences, University of Gondar, Gondar, Ethiopia; Department of Biochemistry, University of Gondar, Gondar, Ethiopia; Department of Biology, Natural and Computational Sciences, Debre Markos University, Debre Markos, Ethiopia; Department of Biology, Natural and Computational Sciences, University of Gondar, Gondar, Ethiopia; Institute of Virology, University of Leipzig, Leipzig, Germany

**Keywords:** Dacryocystitis, Antibiotic resistance, Ethiopia

## Abstract

**Background:**

Bacterial pathogens isolated from dacryocystitis patients are diverse and complex in terms of their distribution, prevalence, and antimicrobial susceptibility pattern. The clinical importance of microbial causes of dacryocystitis and pattern of drug resistance has not been reported in northwest Ethiopia. Moreover, the management of dacryocystitis is based on only clinical observation Therefore, this study attempted to identify and define clinical and microbiological characteristics of microbial agents of dacryocystitis and its antibiotic susceptibility patterns.

**Methods:**

A cross sectional study was conducted from January 2011-January 2012 among dacryocystitis patients attending ophthalmology outpatient department of Gondar University teaching Hospital. Sociodemographic and clinical data collection, microbiological analysis and antibiotic susceptibility test patterns were done following standard procedures.

**Results:**

From the total of 51 dacryocystitis cases, bacterial origins were isolated among 31(60.8%) cases. The dominant isolates were Coagulase negative Staphylococci (CNS) 9(29.0%), *Staphylococcus aureus* (*S. aureus*) 6(19.4%), and *Pseudomonas* species 3(9.7%). *S. pneumoniae*, *Entrobacter* species, *K. pnemoniae* and *H. influenzae* were each accounted 6.5% isolation rate. Among the commonly prescribed antimicrobials tested for susceptibility pattern; amoxicillin 38.7%, ciprofloxacin 25.8%, chloramphinicol 25.8%, co-trimoxazole 25.8%, and ampicillin 19.4% were resistant to the overall bacterial isolates identified. Only *Citrobacter* species were sensitive to all antibiotics tested but the rest bacterial isolates were resistant for at least to one, two, three, four and more antibiotics tested. Overall, 9(29.0%) of the bacterial isolates were resistant to only one antibiotics and resistance to two, three and four antibiotics each accounted 5(16.1%) rate.

**Conclusions:**

Though the information derived from this study was very meaningful, further studies encompassing viral, fungal, parasitic and anaerobic bacterial origin are important to better define the spectrum and relative incidence of pathogens causing dacryocystitis. Microbiological analysis and antimicrobial susceptibility pattern is mandatory for the selection of a specific antimicrobial therapy and to the control of further resistance development of bacterial strains.

## Background

Dacryocystitis is an infection of the nasolacrimal sac of an eye, frequently caused by nasolacrimal duct obstruction. It may be related to a malformation of the tear duct, injury, eye infection, or trauma. Major clinical syndromes are pain, redness, and swelling over the inner aspect of the lower eyelid and epiphora and results in congestion of lacrimal fluid [1]. This further leads to thickening, accumulation of germs and subsequent severe inflammation. Dacryocystitis occurs in acute and chronic forms [2]. The acute form could be associated with severe morbidity and primarily related to the lacrimial sac abscess and spread of infection [2,3]. It usually presents as a preseptal infection, but can uncommonly be associated with orbital cellulitis [3,4]. The chronic form of dacryocystitis is associated with chronic tearing, and conjunctival inflammation and infection [5].

The microbiology of dacryocystitis may differ in its acute and chronic infections. In severe acute dacryocystitis; single infection may predominate, often involving gram-negative rods. Several other species of bacteria could be also involved in the pathogenesis of chronic dacryocystitis. Usually, the majority of patients harbour multiple microorganisms [1,6]. In most cases of dacryocytitis polymicrobial infections were common and concurrently isolated from bacterial, fungal, and viral origin [7,8]. Regarding bacterial origin, however, generally gram-positive organisms were most common [9-11] which were followed by Gram-negative bacteria of both anaerobic and aerobic origin. In particular, most reports showed that fungal pathogens like *Fusarium* spp*.*, *Aspergillus* spp., and *Candida albicans* were the predominant ones isolated in dacroyocystitis patients with other bacterial pathogens [7,8].

The antibiotic treatment for dacryocystitis is dependent on age of the patient, status of the diseases, and the causes of the infection and drug resistance pattern. Especially, the pattern and magnitude of antibacterial resistance may differ from region to region which is highly dependent upon the types of resistant bacterial strains distribution and antimicrobial agents used [12].

The clinical importance of microbial complications of dacryocystitis and drug resistance patterns have not been much reported in Ethiopia. As a result, management of dacryocystitis is based on clinical observation only which has not been supported by microbiological analysis. Clinical presentations and empirical therapy alone are not sufficient as a means of diagnostic and treatment strategy. Therefore, this study attempted to identify and define clinical and bacteriological characteristics of bacterial agents of dacryocystitis and its antibiotic susceptibility patterns in patients attending University of Gondar Eye Specialized Hospital, Northwest Ethiopia.

## Methods

### Study design, period, and area

A cross sectional study was conducted from January 2011-January 2012 among dacryocystitis diagnosed patients attending at Ophthalmology outpatient Department of University of Gondar teaching hospital, Northwest Ethiopia.

### Study population and sample size determination

All consecutive patients with dacryocystitis visiting the hospital in the study period were included and thus a total of 51 dacryocystitis cases were eligible for microbiological analysis.

### Data collection

Sociodemographic data and relevant clinical evaluation of the patients were collected using a structured and pre-tested questionnaire. Standard operating procedures were first prepared to standardize the procedure and to follow the same procedures during sample collection. Patients were first examined by an ophthalmologist and dacryocystitis was clinically defined, and thus specimens were collected from those patients presented dacryocystitis in their nasolacrimal discharge. Specimens were collected with soft-tipped applicators of sterile cotton swabs. Great care was taken to avoid possible contamination of the specimen.

### Microbiological analysis

Inoculation of specimens was done following standard procedures. Specimens were inoculated on blood agar (Oxiod, Hampshire, UK), MacConkey agar (Oxiod, Hampshire, UK) and Chocolate agar (Oxiod, Hampshire, UK) [13].

### Bacterial identification

Bacteria were further characterized and confirmed using colony morphology and their biochemical reactions using the standard biochemical tests.

### Antibiotic susceptibility pattern

Antimicrobial susceptibility testing was performed following the disk diffusion technique [14]. The following antibiotic disks were used; ampicillin (10 μg), chloramphenicol (30 μg), gentamicin (10 μg), vancomycin (30 μg), tetracycline (30 μg), co-trimoxazole (25 μg), amoxicillin (20 μg), ciprofloxacin (30 μg), ceftriaxone (30 μg), erythromycin (15 μg), penicillin (10 μg), and methicillin (5 μg) (Oxiod, Hampshire, UK).

After obtaining a pure culture, a loopful of bacteria was taken from the colony and transferred to a tube containing 5 ml tryptone broth and mixed gently until a homogenous suspension was formed. The turbidity of the suspension was adjusted to the optical density of a McFarland 0.5 tube (0.14-0.15 nm) in order to standardize the inoculum size [13]. The inoculum of each isolate was swabbed onto a Mueller-Hinton (Oxiod, Hampshire, UK), chocolate, and blood agars, depending on the type of bacteria isolated. Antibiotic sensitivity discs were added after drying the plates for 3–5 minutes. Consequently, the plates were incubated aerobically at 35-37°C for up to 48 hours. The diameters of zone of inhibition were measured in millimeter (mm) using callipers and were interpreted as susceptible, intermediate, and resistant according to the Clinical and Laboratory Standards Institute (CLSI) [13]. Reference strains of *S.aureus* (ATCC 25923) and *E. coli* (ATCC25922) were routinely used for a test controls. The culture media sterility was checked for each new prepared media by incubating at 35-37°C for 24 hrs.

### Data analysis

The data were entered and analyzed using SPSS version 16 statistical software program. Pearson Chi-square (*X*^2^) statistics was carried out to see the association of sociodemographic and clinical data to the isolated microbial pathogens. Simple descriptive statistics was also used to explain antibiotic resistance/sensitivity patterns. The intermediate susceptibility test readings were considered as resistance.

### Ethical consideration

Consent was obtained from each patients and ethical approval for the study was obtained from the Research and Community Services Office of the University of Gondar. Microbiological and susceptibility test results were communicated to the physicians for better management of the patients.

## Results

### Patients’ demography

Nasolacrimal discharges was taken from 51 dacryocystitis patients. Among these, 32 (62.7%) were females and the age distribution of the patients ranged from 0.5 to 85 years old with the median age of 53 years. The majority (64.7%) of the patients were adults greater than 30 years old. Most of the attendants came from the surrounding rural areas of Gondar city 17(33.3%) and Wegera-Dabat-Debark route 16(31.4%). Majority 44(86.3%) of them were engaged in agricultural and related activities (Table 1).

**Table 1:** **Distribution of patients investigated for dacryocystitis by sex and age group at University of Gondar Eye Specialized Hospital, Northwest Ethiopia, Gondar, Ethiopia from 2011 to 2012**

**Variables**	**Frequency, n (%)**				
	**Sex**		**Total**	***X*** ^**2**^	**P-value**
	**Male**	**Female**			
**Age in years**				7.28	0.51
≤15	4 (30.8)	9(69.2)	13(100)		
16-30	1(20.0)	4(80.0)	3(100)		
31-45	4(28.6)	10(71.4)	14(100)		
46-60	3(42.9)	4(57.1)	7(100)		
>61	7 (58.3)	5(41.7)	12(100)		
Total	19(37.3)	32(62.7)	51(100)		
**Patients’ address**				6.85	0.74
Gondar city & its vicinity	7(41.2)	10(58.8)	17(100)		
Dembia-Alefa & Chilga-Metema route	2(66.7)	1(33.3)	3(100)		
Tikildingay-Dansha-Humera route	1(25.06)	3(75.0)	4(100)		
Wegera-Dabat-Debark route	5(31.2)	11(68.8)	16(100)		
D/Tabor town & its vicinity	4 (50.0)	4(50.0)	8(100)		
Maksegnit-Belesa route	0(0.0)	3 (100.0)	3(100)		
Total	19(37.3)	32(62.7)	51(100)		
**Occupation**				4.82	0.09
Agricultural based	19(43.2)	25(56.8)	44(100)		
Non-agricultural based	0(0.0)	7(100)	7(100)		
Total	19(37.3)	32(62.7)	51(100)		
**History of chronic illness**				0.22	0.90
Yes	1(50.0)	1(50.0)	2(100)		
No	18(36.7)	31(63.3)	49(100)		
Total	19(37.3)	32(62.7)	51(100)		
**Previous antibiotic use**				1.44	0.49
Yes	8(44.4)	10(55.6)	18(100)		
No	11(33.3)	22(66.7)	33(100)		
Total	19(37.3)	32(62.7)	51(100)		
**Duration of symptoms**				12.57	0.05
Weeks	4(66.7)	2(33.3)	6(100)		
Months	5(62.50)	3(37.5)	8(100)		
Years	7(43.8)	9(56.2)	16(100)		
I don’t know	3(14.3)	18(85.7)	21(100)		
Total	19(37.3)	32(62.7)	51(100)		
**Trauma**				4.86	0.09
Yes	4 (80.0)	1(20.0)	5(100)		
No	15(32.7)	31(67.4)	46(100)		
Total	19(37.3)	32(62.7)	51(100)		
**Dacryocysitis**				0.89	0.58
Acute	5 (35.7)	9(64.3)	14(100)		
Chronic	14 (37.8)	23(62.2)	37(100)		
Total	19(37.3)	32(62.7)	51(100)		

### Bacterial isolates

Among 51 dacryocystitis cases, 14(27.5%) and 37(72.5%) had acute and chronic manifestation, respectively. Bacterial origins were isolated among 31(60.8%) cases which were positive for different types of bacterial pathogens. The dominant isolates were from Gram positive groups; CNS 9(29.0%) and *S .aureus* 6(19.4%). From Gram-negatives group, *Pseudomonas* species 3(9.7%) was common. The *S. pnemoniae, Entrobacter* species, *K. pnemoniae* and *H. influenzae* were each accounted for 2(6.5% isolation rate (Table 2). Among the total of 31 bacterial isolates with dacryocystitis, 22 (71.0%) of them were isolated from female patients.

**Table 2:** **Distribution of bacterial agents isolated from dacryocysitis infection among sex at University of Gondar Eye Specialized Hospital, Northwest Ethiopia, Gondar, Ethiopia from 2011 to 2012**

	**Frequency, n (%)**		
	**Sex**		**Total**
**Variables**	**Male**	**Female**	
**Gram positive bacteria**			
*S .aureus*	1 (16.7)	5(83.3)	6(11.8)
CNS*	4 (44.4)	5(55.6)	9(17.6)
*S. pneumoniae*	0 (0.0)	2(100.0)	2(3.9)
*Entrobacter* species	0 (0.0)	2(100.0)	2(3.9)
***Gram negative bacteria***			
*E .coli*	0 (0.0)	1(100.0)	1(2.0)
*K. pneumoniae*	1(50.0)	1(50.0)	2(3.9)
*Pseudomonas* species	0 (0.0)	3(100.0)	3(5.9)
*Citrobacter* species	0 (0.0)	1(100)	1(2.0)
*H.influenzae*	2(100.0)	0(0.0)	2(3.9)
*P.vulgaris*	0 (0.0)	1(100)	1(2.0)
*P. mirabilis*	0 (0.0)	1(100)	1(2.0)
*Providencia* spp	1(100.0)	0 (0.0)	1(2.0)
**Total**	9(29.0)	22(71.0)	31(60.8)**

*CNS-Coagulase Negative *Staphylococci*.

**Among the total of 51 dacryocystitis cases.

### Antimicrobial susceptibility test

The commonly prescribed antimicrobials were tested for its susceptibility pattern; amoxicillin 38.7%, ciprofloxacin 25.8%, chloramphinicol 25.8%, co-trimoxazole 25.8%, and ampicillin 19.4% were resistant to the overall bacterial isolates identified. Among gram positives; CNS showed high multiple antibiotic resistance rates for ampicillin 4(44.4%), where as penicillin and co-trimoxazole each accounted for 3(33.3%) resistance rate. Similarly, *S.aureus* showed highest multiple antibiotic resistances for erythromycin, penicillin, and co-trimoxazole each accounted for 2 (33.3%) and tetracycline alone accounted for 3 (50.0%) resistance rates. *S. pneumoniae* showed complete (100%) resistance for ampcillin and tetracycline. Similarly *Entrobacter* species showed complete resistance for nalidic acid and co-trimoxazole (Table 3).

**Table 3:** **Antimicrobial susceptibility test patterns of bacterial pathogens isolated from dacryocystitis infection, University of Gondar Eye Specialized Hospital, Northwest Ethiopia, Gondar, Ethiopia from 2011 to 2012**

***Bacterial*** **isolates**	**No. (%)**	**Pattern**	**Common antibiotics tested, No. (%)**											
			**AMP**	**AMOX**	**CIP**	CN*	MET*	**NA**	**CAF**	ERY*	PEN*	**TTC**	**SXT**	**CEF**
*S .aureus*	6(19.4)	S	5(83.3)	5(83.3)	6(100.0)	-	5(83.3)	6(100.0)	6(100.0)	4(66.7)	4(66.7)	3(50.0)	4(66.7)	6(100.0)
		R	1(16.7)	1(16.7)	0(0.0)	-	1(16.7)	0(0.0)	0(0.0)	2(33.3)	2(33.3)	3(50.0)	2(33.3)	0(0.0)
CNS	9(29.0)	S	9(100.0)	7(77.8)	5(55.6)	-	9(100.0)	9(100.0)	7(77.8)	9(100)	6(66.7)	7(77.8)	6(66.7)	8(88.9)
		R	0(0.0)	2(22.2)	4(44.4)	-	0(0.0)	0(0.0)	2(22.2)	0(0.0)	3(33.3)	2(22.2)	3(33.3)	1(11.1)
*S. pnemoniae*	2(6.5)	S	0(0.0)	2(100.0)	1(50.0)	-	2(100.0)	2(100.0)	1(50.0)	2(100)	2(100)	0(0.0)	2(100)	2(100)
		R	2(100.0)	0(0.0)	1(50.0)	-	0(0.0)	0(0.0)	1(50.0)	0(0.0)	0(0.0)	2(100.0)	0(0.0)	0(0.0)
*Entrobacter spp.*	2(6.5)	S	2(100.0)	1(50.0)	1(50.0)	-	-	0(0.0)	1(50.0)	1(50.0)	1(50.0)	2(100.)	0(0.0)	2(100)
		R	0(0.0)	1(50.0)	1(50.0)	-	-	2(100.0)	1(50.0)	1(50.0)	1(50.0)	0(0.0)	2(100)	0(0.0)
*E .coli*	1(3.2)	S	1(100.0)	0(0.0)	1(100.0)	1(100.0)	-	0(0.0)	1(100.0)	-	-	0(0.0)	1(100)	1(100)
		R	0(0.0)	1(100)	0(0.0)	0(0.0)	-	1(100.0)	0(0.0)	-	-	1(100.0)	0(0.0)	0(0.0)
*K. pnemoniae*	2(6.5)	S	1(50.0)	1(50.0)	2(100.0)	2(100.0)	-	1(50.0)	2(100.0)	-	-	1(50.0)	2(100.)	1(50.0)
		R	1(50.0)	1(50.0)	0(0.0)	0(0.0)	-	1(50.0)	0(0.0)	-	-	1(50.0)	0(0.0)	1(50.0)
*Pseudomonas* spp.	3(9.7)	S	2(66.7)	1(33.3)	2(66.7)	2(66.7)	-	2(66.7)	2(66.7)	-	-	2(66.7)	2(66.7)	3(100.)
		R	1(33.3)	2(66.7)	1(33.3)	1(33.3)	-	1(33.3)	1(33.3)	-	-	1(33.3)	1(33.3)	0(0.0)
*Citrobacter* spp.	1(3.2)	S	1(100.0)	1(100.0)	1(100.0)	1(100.0)	-	1(100.0)	1(100.0)	-	-	1(100)	1(100)	1(100)
		R	0(0.0)	0(0.0)	0(0.0)	0(0.0)	-	0(0.0)	0(0.0)	-	-	0(0.0)	0(0.0)	0(0.0)
*H.influenzae*	2(6.5)	S	2(100.0)	0(0.0)	2(100.0)	2(100.0)	-	2(100.0)	1(50.0)	-	-	1(50.0)	1(50.0)	2(100.0)
		R	0(0.0)	2(100)	0(0.0)	0(0.0)	-	0(0.0)	1(50.0)	-	-	1(50.0)	1(50.0)	0(0.0)
*P.vulgaris*	1(3.2)	S	1(100.0)	0(0.0)	1(100.0)	1(100.0)	-	1(100.0)	1(100.0)	-	-	1(100)	1(100)	1(100)
		R	0(0.0)	1(100)	0(0.0)	0(0.0)	-	0(0.0)	0(0.0)	-	-	0(0.0)	0(0.0)	0(0.0)
*P. mirabilis*	1(3.2)	S	0(0.0)	0(0.0)	1(100.0)	1(100.0)	-	1(100.0)	0(0.0)	-	-	1(100.0)	1(100)	1(100)
		R	1(100)	1(100)	0(0.0)	0(0.0)	-	0(0.0)	1(100)	-	-	0(0.0)	0(0.0)	0(0.0)
*Providencia* spp	1(3.2)	S	1(100.0)	0(0.0)	1(100.0)	0(0.0)	-	1(100.0)	1(100.0)	-	-	0(0.0)	0(0.0)	1(100)
		R	0(0.0)	1(100)	0(0.0)	1(100)	-	0(0.0)	0(0.0)	-	-	1(100)	1(100)	0(0.0)
**Total**	31(100)	S	25(80.6)	19(61.3)	23(74.2)	10(83.3)	17(89.5)	27(87.1)	23(74.2)	16(84.2)	13(68.4)	22(71.0)	23(74.2)	29(93.5)
		R	6(19.4)	12(38.7)	8(25.8)	2(16.7)	2(10.5)	4(12.9)	8(25.8)	3(15.8)	6(31.6)	6(29.0)	8(25.8)	2(6.5)

Keys: AMP- Ampicillin, AMOX-Amoxicillin, CIP- Ciprofloxacin, CN- Gentamycin, MET- Methicillin, NA-Nalidic Acid, CAF-Chloramphinicol, ERY- Erythromycin, VAN- Vancomycin, PEN-penicillin, TTC- Tetracycline, SXT- Co-trimoxazole, CEF-Ceftriaxone. *Percentage calculation is based on column total [19] denominator.

Among the Gram negative bacteria; *E. coli (*for amoxicillin, nalidic acid and tetracycline), *Psuedomans* species and *H. influnzae* (for amoxicillin), *P. mirabilis (*for amoxicillin and co-trimoxazole) and *Providencia* species (for amoxicillin, gentamicin, tetracycline and co-trimoxazole) showed complete resistance (Table 3).

Out of 31 bacterial isolates, 28(90.3%) were resistant to multiple antibiotics (resistant for two and more antibiotics) (Table 4). Among the isolates, CNS showed resistance to four antibiotics isolated. *S. aureus* was resistant to three antibiotics. *S. pnemoniae, Entrobacter* species, *K. pnemoniae, P. arugenosia, and H. influenzae,* each was resistant to two antibiotics. *E .coli*, *P.vulgaris, P. mirabilis, and Providencia* species were each resistant to one antibiotics tested. Only *Citrobacter* species were sensitive to all antibiotics tested. The rest bacterial isolates were resistant for at least to one, two, three, four, and more antibiotics tested (Table 4). Overall, 9(29.0%) of the bacterial isolates were resistant to only one antibiotics and resistance to two, three, and four antibiotic each accounted for 5(16.1%) rate. Four (12.9%) of them showed resistance to 5 antibiotics tested (Table 4).

**Table 4:** **Multiple antibiotic resistance patterns of bacterial pathogens isolated from dacryocystitis infection, University of Gondar Eye Specialized Hospital, Northwest Ethiopia, Gondar, Ethiopia from 2011 to 2012**

**Isolates**	**No (%)**	**Antibiotic resistance patterns**					
		**Ro**	**R1**	**R2**	**R3**	**R4**	**R5**
*S .aureus*	6(19.4)	1(16.7)	3(50.0)	1(16.7)	-	-	1(16.7)
CNS	9(29.0)	1 (11.1)	4(44.4)	1(11.1)	1(11.1)	-	2(22.2)
*S. pnemoniae*	2(6.5)	-	-	1(50.0)		1(50.0)	-
*Entrobacter species*	2(6.5)	-	-	-		1(50.0)	1(50.0)
*E .coli*	1(3.2)	-	-	-	1(100.0)	-	-
*K. pnemoniae*	2(6.5)	-	-	1(50.0)	1(50.0)	-	-
*Pseudomonas* spp.	3(9.7)	-	1(33.3)	-	-	2(66.7)	-
*Citrobacter* spp	1(3.2)	1(100.0)	-	-	-	-	-
*H.influenzae*	2(6.5)	-	-	1(50.0)	1(50.0)	-	-
*P.vulgaris*	1(3.2)	-	1(100.0)	-	-	-	-
*P. mirabilis*	1(3.2)	-	-	-	1(100.0)	-	-
*Providencia* spp	1(3.2)	-	-		-	1(100.0)	-
**Total**	31(100)	3 (9.7)	9 (29.0)	5(16.1)	5(16.1)	5(16.1)	4 (12.9)

Keys: R_o_- sensitive to all antibiotics, R1 – resistant to 1 antibiotic.

R_2_- resistant to 2 antibiotics, R3 – resistant to 3 antibiotics.

R_4_- resistant to 4 antibiotics, R_5_ – resistant to 5 and more than 5 antibiotics.

## Discussion

In this study, patients with adulthood age group and occupationally engaged in agricultural activities were more exposed to dacryocystitis. In line to this study, Gopinathan *et al.* [15] reported that patients with agricultural-based activities were at greater risk of developing microbial ocular infections particularly keratitis and ocular trauma. It is also known that dacryocystitis is common in middle and elderly age and more commonly in women but of course could affects all age groups [16]. The current finding showed that 62.7% of women regardless of their age group and median age of above 53 years old regardless of their sex had dacryocystitis. Both acute and chronic manifestations of dacryocystitis were also evident in this study which is in line to several similar studies [2,3,17,18] which reported both clinical phases of dacryocystitis.

The presentation of dacryocystitis can also vary according to geographical area and the microbiological aetiology [19]. The microbiological variation in our study was so evident that we could isolate 12 different species of bacteria. As it has been also common in many of similar studies [18,20] which focused on the microbiological analysis of the etiologic agents of the dacryocystitis, bacterial origins were accounted for 60.8% of the isolates of the current study. Gram positive bacteria accounted for higher isolation rate (61.3% vs 38.7%) with comparability of Kebede *et al*. [21] reported in Addis Ababa, Ethiopia which showed 62.6% Gram positives and 37.4% Gram negatives. Interestingly, the current findings and previous reports by Kebede *et al*. [21] were also in line with most similar studies [6,8,12,18,20,22]. For instance, 62% in Finland [16], 54.4% in Australia [7], 79.1% in Saudi Arabia [23], 71.2% in Iran [24] and 68.8% in USA [18] accounted for Gram positive bacterial origin in dacryocystitis infection.

In addition, the magnitude of Gram positive isolates (29.0% CNS, 19.4% *S. aureus*, 3.9% *S. pneumoniae,* and 3.9% of *Entrobacter* species) was more or less comparable with other similar studies [21,25,26] in terms of frequency of isolation. Similarly, the Gram negative isolates with the predominate *Pseudomonas* species (9.7%) followed by *K. pneumoniae* (6.5) and *H. influenzae* were equally accounted for 6.5% isolation rate. In regard to prevalence of each bacterium, however, it was obvious that there existed discrepant reports. For instance, the predominance of *Staphylococcus* species from gram-positive groups and *H. influenzae* from gram-negative groups followed by *Pseudomonas*, *Proteus* and *Citrobacter* spps.were reported elsewhere (6, 8 9, 10, 16). Another study reported a predominant isolate of CNS (62.1%), followed by *S. aureus* (20.7%) and *S. pneumoniae* (17.2%) [18]. Grant [5] also reported that the most common organisms isolated from the lacrimal sacs with dacryocystitis included *S.aureus*, *S. epidermids, Streptococcus, Pseudomonas,* and *Pneumococcus* species [5]*.*

Since anaerobic bacterial groups were not considered in this study, concluding gram-negatives accounted for a lower isolation rate might be misleading and as the result the current findings must be interpreted with caution. Nevertheless, many of similar studies [20,22,23,27] dealing about microbiological profile of dacryocystitis to the full spectrum of bacterial (all forms), fungal, parasitic and viral origins confirmed that Gram positive bacteria were the predominant isolates [20]. In general, Gram-positive groups were reported in many studies as the predominant group which were followed by Gram negatives with great variations in prevalence rates and isolate types. The source of such general variation could be associated to the number of dacryocystitis cases by itself, patients’ sociodemographic and geographical differences, access to eye care, and practices to public health awareness.

Dacryocystitis with whatsoever causative agents, usually results in blockage of the nasolacrimal duct. The treatment of such obstruction is surgical intervention. However, there is a fivefold risk of soft tissue infection after open lacrimal surgery without systemic antibiotic prophylaxis that represents a significant risk of failure in lacrimal surgery [21]. This showed that the role of antibiotics as a therapeutic options as well as prophylaxis agent for the management of dacryocystitis is very important. Nevertheless, the emergence of antibiotic resistance becomes a challenge not only for dacryocystitis management but also for any sort of clinical practices where antibiotics are necessary [28].

In this study, the antimicrobial susceptibility tests revealed that ceftriaxone (95.3%), erythromycin (84.2%), nalidic acid (87.1%), gentamycin (83.3%) were more effective than other antibiotics tested to all bacterial isolates. In Kebede et al. report [21] the antibiotics to which the majority of the isolates sensitive were chloramphenicol (82.4%), gentamycin (79.1%), erythromycin (68.1%), and tetracycline (61.5%).

In the current study, *S. aureus* was sensitive to all ciprofloxacin, nalidic acid, chloramphenicol, and ceftriaxone. Similarly, all isolates of CNS were sensitive to ampicillin, methicillin, nalidic acid, and erythromycin. Almost one-third (33.3%) of the isolates of *S. aureus* were resistant to erythromycin, penicillin, tetracycline, and co-trimoxazole. Similar resistance patterns were observed to ciprofloxacin, penicillin, and co-trimoxazole for CNS. An expectedly, a single isolate of *S. aureus* was resistant to methicillin. As a result, the emergence of methicillin resistance *S. aureus* in this teaching hospital may pose therapeutic problems, and therefore the empirical antibiotic treatment should be avoided and rather performing antimicrobial susceptibility testing is highly needed. Moreover*, S. pneumoniae* was completely resistant to tetracycline and ampicillin and 50% resistant rate to chloramphenicol was in contrast to earlier finding by Kebede et al. [21] which showed 95.2% and 100% of *S. pneumoniae were* sensitive to chloramphenicol and tetracycline, respectively. The observed increase in drug resistance rate to chloramphenicol and tetracycline was not surprising, since these antibiotics were the most commonly used antibiotics to treat infections empirically in the study area. Moreover, indiscriminate use of antibiotics both within the hospital and the community at large were also another imposing factor which played its own role for the problem.

From the dominant gram-negative isolates; *K. pneumonae*, *Pseudomonas* species, and *H.* influenzae were resistant to most antibiotics tested which were higher than previous study done by Kebede et al. [21]. Chloramphenicol, a commonly prescribed antibiotic showed 74.2% sensitivity patterns to all isolates tested which was far less than other similar study [27] which reported 100% sensitive rates. This might be a reflection of inappropriate use of antibiotics, lack of diagnostic laboratory services and unavailability of guideline regarding the selection of drugs that enforce to empirical treatment options.

Taken the findings of this study in to account, the presence of higher percentage of single and multiple antibiotic resistance patterns were common in the area. Most of the times, in our set up broad spectrum antimicrobial agents were empirically prescribed to treat bacterial infections without definite diagnosis. In the community side, irrational use of antibiotics was also a common practice. For instance, in this study 35.3% of the dacryocystitis patients had a history of antibiotic usage of any sort before they attended the hospital for their dacryocystitis management. It was also obvious that such practices are fuelling the already existing natural antibiotic resistance mechanisms of bacteria and might be responsible for the relatively higher prevalence rate of resistance to amoxicillin, ciprofloxacin, chloramphenicol, co-trimoxazole, and ampicillin in this study. Due to facility limitations, isolation of *Chlamydia trachomatis,* anaerobic bacteria, potential fungal and viral causative agents were not considered.

## Conclusion

The current study showed that bacterial isolates from dacryocystis patients were diverse in terms of their distribution, prevalence, and pattern of antimicrobial drug resistance and/or sensitivity. Thus the current findings were very meaningful so as to optimize antibiotic prophylaxis and treatment options in our setup. We recommend that the antimicrobial susceptibility testing should be done regularly as part of the routine clinical and diagnostic practices to manage the dynamic nature of resistance patterns of microbial infections over time.
